# Blood donors deferral prevalence and causes in a tertiary health care hospital, southern Nigeria

**DOI:** 10.1186/s12913-019-4352-2

**Published:** 2019-07-22

**Authors:** Henshaw Uchechi Okoroiwu, Enosakhare Aiyudubie Asemota

**Affiliations:** Department of Medical Laboratory Science, Haematology Unit, University of Calabar, Calabar, Nigeria

**Keywords:** Donor deferral, Deferral pattern, Donor selection, Donor selection criteria, Blood donation

## Abstract

**Background:**

Blood transfusion is a life-saving intervention. However, the safety of the donor and the recipient is paramount. This study was aimed at determining the blood donation deferral pattern of University of Calabar Teaching Hospital.

**Methods:**

A retrospective analysis of the prospective donors’ data of University of Calabar Donor clinic within the period of March 2015 to February 2016 was conducted. Data were extracted from the donor register and analyzed. Prospective donors were screened and interviewed for causes of temporary and permanent deferrals.

**Result:**

Out of the 1886 screened prospective donors, 164 (8.69%) were deferred. Though the minority of the donor population, female donors had the highest deferral rate (33.33%). There were 31.10 and 68.90% cases of temporary and permanent deferrals, respectively. Hepatitis B virus (HBV) was the highest (31.71%) cause of overall deferral as well as permanent deferral. Anemia was the major (21.95%) cause of temporary deferral as well as the second cause of overall deferrals. Commercial and replacement donors constituted 68.28 and 31.71% of the deferral cases, respectively.

**Conclusion:**

HBV was found to be the overall leading cause of deferral in the studied area. This outcome poses a public health concern and should elicit measures to curb the infection rate.

## Background

Blood transfusion is a life-saving intervention and plays important role in medical and surgical practice [1]. An efficient blood transfusion service is critical to good health care delivery [2]. Despite the life-saving role of blood transfusion, the role of blood transfusion services is to ensure adequate availability of safe blood via donors that are in good health [3, 4]. Although adequate supply of blood is paramount in blood transfusion practice, the caveat is to ensure the collection and transfusion protocol do not endanger both the recipient and the donor [3]. This is actualized by deriving donor deferral criteria [5], strict adherence to screening of prospective donors for transfusion transmissible infections [6, 7] and other risk behaviors. Deferrals could be temporary or permanent [8]. Temporary deferral connotes that the prospective donor is deferred based on removable, time bound factor such as low hemoglobin, hematocrit and more, while permanent deferral implies that the prospective donor have non-removable, long lasting factor such as possibility for any of the transfusion-transmissible infections (TTIs) [2].

All deferred donors are expected to be treated with respect and care in a confidential manner and should be given a clear explanation of the reason for the deferral and an opportunity to ask questions [8]. Though not specific, the general guideline for prospective donor selection and deferral in Nigeria is contained in Nigerian National Blood Policy (revised) [9]. Nigeria in December 2006 established a national blood transfusion policy that culminated to national blood transfusion practice. The policy is made up of series of themes that are directed towards constant supply of safe and affordable blood donor units. The Nigerian national blood policy stratified its services into; National blood transfusion service (NBTS), zonal blood transfusion centers, state and local government service centers, armed forces service centers, private and other nongovernmental health organizations [7, 10]. Current NBTS guideline mandate the screening of all donor units for anti HCV, hepatitis B surface antigen, anti HIV and syphilis using combination of rapid qualitative immunochromatographic test kits, enzyme-linked immunosorbent assays and antigen assays [10].

There are three main classification of donors in Nigeria; the voluntary (non-remunerated) donors, family (replacement) donors and commercial (paid) donors [11, 12]. The voluntary donors are altruistic individuals who donate blood with the sole aim of saving life without regard to any form of inducement. These are usually mobilized through mass media, blood donation drives to churches and schools. The family (replacement) donors includes those that donate for hospitalized family members, friends or associates and could be nonremunerated or remunerated via family recruitment. On the other hand, commercial donors refer to those who donate blood strictly for financial gratification [12]. In Nigeria, commercial donors are remunerated between N2,000 ($5.6 @ $1:N 360) to N10,000 ($27.7 @ 1$:N 360) depending on the location and the blood group type (scarcity of the needed blood group) [Personal observation].

Knowledge of the pattern and reasons for donor deferral is crucial in guiding for future recruitment plans. Hence, this study was aimed at determining the rate and reasons for donor deferral in University of Calabar Teaching Hospital.

## Methods

### Study setting

This study was carried out in the blood bank unit of the University of Calabar Teaching Hospital (UCTH), Calabar, Cross River State, Nigeria, which is the only tertiary health institution in the state. It has 410 bed space capacity. Cross River state is located in southern Nigeria [13] in the delta region. Cross River State has an area of 21,787 km^2^ and a population of 2,892,988 (2006 census) [14]. University of Calabar Teaching Hospital is located in Calabar metropolis within the state with an estimated population of 373,196 (2006 census) suggesting this population to represent about 0.3% of the Nigerian population (140,000,000) (2006 census) [15]. Earlier World Health Organization data on blood availability reported 0.7 units/1000 inhabitants for Nigeria [16].

### Study design

This study took retrospective approach in analyzing data of prospective donors to evaluate the different causes of blood donor deferral in University of Calabar Teaching Hospital from March 2015 to February, 2016.

### Data collection

The data were extracted from the donor register of the Donor Clinic of the University of Calabar Teaching Hospital, Calabar. Within the study period, 1886 prospective donors were screened for anemia (packed cell volume/hematocrit), transfusion transmissible infections (Hepatitis B virus [HBV], Hepatitis C virus [HCV], Human Immunodeficiency Virus [HIV] and syphilis) and other chronic diseases. The donors were within 18–65 years of age and weighed not less than 50 kg.

### Methodology

The prospective donors were screened for transfusion transmissible infections (HBV, HCV, HIV and syphilis) using commercially available kits following the manufacturers’ instruction. The study center used serum/plasma rapid immunochromatographic kits manufactured by ABON Biopharm (Hangzhou) Co. Limited for hepatitis C (HCV) and hepatitis B surface antigen (HBsAg) screening. Treponemal antibody was screened using ACON rapid immunochromatographic VDRL (ACON Laboratories Inc., San Diego, CA, USA). The HIV screening was performed using two kits; Determine HIV 1/2 (Alere Medical Co. Limited Japan) and Uni-gold Recombigen HIV 1/2 (Trinity Biotech, Ireland).

The sensitivity and specificity of the HCV kit were 100 and 86.11%, respectively while the sensitivity and specificity of the HBsAg kit were both 99.0%. The sensitivity and specificity of the VDRL kit were both 98.6%. For the HIV kits, the sensitivity and specificity of the Alere Determine kit were 100.0 and 99.4%, respectively, while the sensitivity and specificity of the Uni-gold recombigen kit were 95.5 and 100.0%, respectively.

Packed cell volume (PCV)/hematocrit was used to assess anemia using microhematocrit method (centrifugation method). A minimum cutoff point of 0.4 L/L (40%) was used in the study centre for both gender. Other chronic diseases and history of previous blood donation were assessed via oral interview. Predonation screening is practiced in the study centre. Prospective donors were bled if they passed the screening and deferred if they didn’t.

### Statistical analysis

Generated data were analyzed using SPSS version 22 (IBM Corps, Armonk, NY, USA). Categorical variables were represented as frequencies and percentages. Fisher exact test and odd ratio were used to assess association between categorical variables. Significant difference was determined at alpha value of 0.05.

## Results

Majority (98.2%, *n* = 1853) of the prospective donors were males. Age range 18–28, 29–38, 39–48, 49–58, and 59–65 years represented 17.15% (*n* = 324), 56.36% (*n* = 1063), 12.14% (*n* = 229), 10.71% (*n* = 202) and 3.61% (*n* = 68) of the prospective blood donors, respectively. The ABO blood group of the prospective blood donors were in the order O > A > B > AB (Table 1).

**Table 1:** Demographic characteristics of the prospective blood donors

Variable	Frequency	Percentage (%)
Gender		
Male	1853	98.25
Female	33	1.75
Age (Years)		
18–28	324	17.15
29–38	1063	56.36
39–48	229	12.14
49–58	202	10.71
59–65	68	3.61
Abo Blood Group		
O	1335	70.78
A	334	17.71
B	209	11.08
AB	8	0.43

Among the 1886 prospective donors who presented themselves within the study period, a total of 164 of the prospective donors were deferred giving a deferral rate of 8.69%. Out of the 1853 males that were screened, 153 were deferred giving deferral rate of 8.26% while 11 of the 33 females screened were deferred giving deferral rate of 33.33%. Male donors were less likely to be deferred with an odd ratio of 0.305 (CI: 0.179–0.520) (Table 2).

**Table 2:** Deferral pattern of prospective blood donors based on gender

Gender	N. screened	N. deferred	Deferral rate (%)	*P*-value	OR	CI
Male	1853	153	8.26	0.000^a^	0.305	0.179–0.520
Female	33	11	33.33		1	
Total	1886	164	8.69			

^a^Fisher exact test

*N* absolute number

There were more (68.90%) cases of permanent deferral than temporary deferral (31.10%). Further stratification showed that Hepatitis B virus (HBV) (31.71%) was the overall highest cause of deferral as well as the commonest cause of permanent deferral. Low packed cell volume/hematocrit (anemia) (21.95%) constituted the second cause of overall deferral as well as the highest cause of temporary deferral. Hepatitis C (HCV), HIV, and syphilis constituted 18.90, 18.29 and 9.15% causes of deferral during the study period (Table 3).

**Table 3:** Causes of deferral among the studied population

Causes of deferral	Number deferred	% of total deferral
Permanent deferral		
HBV	52	31.71
HCV	31	18.90
HIV	30	18.29
Total	113	68.90
Temporary deferral		
Syphilis	15	9.15
Low hemoglobin	36	21.95
Total	51	31.10

Commercial donors (9.27%) and replacement donors (7.87%) represented majority of the deferred cases. Commercial and replacement donors constituted 68.29 and 31.71% of the total deferral. Approximately 7.87 and 9.27% of the replacement and commercial donors screened were deferred. We did not record any deferral of the voluntary donors within the study period. The donor pool was dominated by commercial and replacement donors; 64.05 and 35.05% (Table 4).

**Table 4:** Deferral rate based on type of blood donor

Donor type	No. screened	No. deferred (%)	Proportion of total deferral rate (%)
Voluntary donor	17	0 (0.00)	0.00
Replacement donor	661	52 (7.87)	31.71
Commercial donor	1208	112 (9.27)	68.29

*No* absolute number

## Discussion

Male donors constituted majority (98.2%) of the prospective donors in the present study. This finding is in keeping with previous reports in Nigeria [2, 7, 17], Cameroon [18], Ethiopia [19], India [4, 20], Pakistan [21] and Mexico [22]. This trend has been attributed to cultural dogma that women should abstain from blood donation owing to their monthly blood loss through menstruation [7].

This study observed a deferral rate of 8.69%. This value is quite low when compared to 16 and 32.5% reported in Uyo [23] and Nnewi [2], respectively, being studies in Nigeria. Similar deferral rates as ours have been reported in India (8.83%) [24], Saudi Arabia (8.7%) [25], Pakistan (10.4%) [26]. However, lower deferral values have been reported in India (4.6–5.6%) [3, 4, 27], Saudi Arabia (4.3%) [28], Iran (4.5%) [29]. Conversely, higher deferral values; 12.8% as reported by Zou and colleagues [30] in their 6-year study of deferral pattern of American Red Cross Blood Services and 13.6% as reported by Custer and colleagues [31] in another study in USA and 14.6% as reported by Aslan et al [32] in a study in Turkish population. The differences in the deferral rates is probably due to differences in the donor selection criteria used in the different studies; lack of scientific and accurate cut-off of hemoglobin, lack of compulsory blood screening for TTls, donation intervals, high risk sexual activities and prevailing medical and endemic conditions [27]. More strict donor eligibility criteria and the level of keenness of the blood donor counsellors on exploring risk behaviors to clients may lead to increased rate of deferral [33]. Socio-economic differences among countries may also contribute to the variation as middle and high income countries may have lower prevalence of infectious diseases and higher nutritional status, and consequently higher hemoglobin (hematocrit) as opposed to low income countries [34, 35]. The World Health Organization mandates every country to have a national blood policy that defines recruitment, selection, deferral, blood screening for transfusion transmissible infections (TTIs), confirmatory testing donor notification, testing and referral [8].

Gender stratification of the deferred donors showed that females had significantly higher deferral rate (33.33%) than their male counterparts (8.26%) with the males having odds of 0.305 against 1 to be deferred. This trend is well documented in previous studies in Nnewi, Nigeria [23] and India [3, 36]. Higher deferral rate in women due to anemia has been documented in the study region [23] and in other developing countries [37].

Commercial and replacement donors constituted majority of the deferred cases; both contributed to 68.29 and 31.71%, respectively of the deferred donors. This finding is in consonance with previous findings [7, 33, 38, 39] where replacement donors were deferred more than non-remunerated (voluntary) donors. The implication of this is that voluntary donors are relatively safer than replacement or commercial donors which corroborate the World Health Organization (WHO) recommendation of 100% voluntary blood donation [40]. The trend of donor type observed in this study is well documented in Nigeria [7, 17, 41]. Voluntary blood donors represented a very small fraction (0.9%) of the donors in this study. Despite the 43 and 10% voluntary nonremunerated blood donation reported in 2013 and 2018, respectively for Nigeria [16, 42], commercial and replacement donation is predominant in most healthcare institutions across the country. The data that generated the 2013 report value was computed from approximately 10% of the general blood collection centers. Owing to logistics and organizational challenges associated with national blood transfusion service in Nigeria, the amount of voluntarily donated blood units have continued to fall over the years, paving way for commercial and replacement blood donations to become the predominant practice [17, 41]. Commercial remunerated donation is the norm especially in hospital based blood banks. It is pertinent to note that the “practical” difference between family replacement donors and commercial donors is grey. Most times, patients’ care givers recruit and present commercial donors as replacement donors [40]. There is need for political willingness on the part of Nigerian government to design and implement workable policies that will boost voluntary donations. Huis in ‘t Veld and colleagues [43] advocated for more tailored, effective and personalized recruitment strategies and channels such as social media while focusing on motivation and demotivation to boost voluntary blood donation..

Permanent deferral represented a large part (68.90%) of the deferral cases. This observation is comparable to the report of Ekwere and colleagues [23]. However, it is at variance with the report of Valerian et al [33] and Rehman et al [20]. The reason for this disparity may not be far-fetched. The present study consisted mostly of remunerated (replacement/commercial) donors while the study by Valerian and colleagues consisted of 90% voluntary non-remunerated donors. Family/replacement and commercial donors have been reported to conceal their actual health status and lifestyle for the sake of saving the life of a loved one and the financial benefits, respectively [40]. Thus, this might have caused lack of relevant information of prospective donors that could lead to temporary deferral except the Hb/hematocrit value that does not need self-report.

The present study has shown that transfusion transmissible infections (TTIs) constituted much (78.05%) of the deferral cases. On individual probe, we observed that HBV was the major cause of deferral (31.71%) as well as the leading cause of permanent deferral (40.63%) followed by HCV and HIV. Previous studies [3, 7, 20] have indicated similar trends. The national survey in Nigeria showed similar trend of higher prevalence of hepatitis B (12.2%) [44] than HIV [45]. Decline of HIV seroprevalence has been reported [46, 47]. This has been credited with the several intervention programmes geared towards HIV over the years; PEPFAR [48] FHI 360 [49] and NACA [50]. Conversely, there is low awareness of HBV, the causative agent, mode of transmission and its health consequences when compared to HIV [33]. The trend of deferral based on TTIs in this study partly mirrors the prevalence of the TTIs in the general Nigerian population. Even in the general population of Nigerians, the prevalence of HBV is greater than that of HIV. Earlier World Health Organization (WHO) report had estimated HBV and HCV prevalence of 11.2 and 2.0%, respectively [51], while HIV prevalence (WHO/UNAIDS) is estimated at 2.8% [52, 53]

Anemia (low packed cell volume/hematocrit) was the major cause of temporary deferral (followed by syphilis) as well as the second leading cause of all deferral in the studied population. Similar finding has been reported in previous studies [2, 20, 23, 33, 54]. The implication of this finding is a high prevalence of anemia in the studied population. This could be as a result of poor nutritional status (inadequate consumption of iron containing diet, folic acid and vitamin B12) [55, 56], concurrent tropical disease, hookworm infection and more. Considering the fact that majority of the donors were commercial and replacement donors, previous donations may also be a contributing factor in the high deferral rate due to hemoglobin. Remunerated donors have been reported to conceal their donation history when they are in desperate need of money [57]. Earlier study has shown that regular blood donors are at the risk of developing depletion of iron from the iron stores [58]. One unit of blood donation has been shown to result in depletion of up to 236 mg of iron from iron stores [58, 59].

Comparatively, the overall deferral based on anemia (*n* = 36/1886; 1.9%) in this study is low when compared to other previous studies in Nigeria; 25.3% in a study in Nnewi southeastern Nigeria by Aneke and colleagues [2] and 6.2% in a study in Uyo southern Nigeria by Ekwere et al [23]. However, the finding is similar to 1.4, 2.86 and 2.24% being studies in India [20], Tanzania [33] and India [58], respectively. Several factors ranging from methodology and demographic factors can explain these variations. While the present study used PCV/hematocrit cutoff of 0.4 L/L (40%) using microhematocrit method, the study by Aneke and colleagues [2] used hematocrit and hemoglobin cutoff of 38% and 12.5 g/dL, respectively without specifying the method used. Ekwere and colleagues [23] used hemoglobin cutoff of 12.5 g/dL without specifying the method used while the study by Rehman et al [20] used hemoglobin cutoff of 12.5 g/dL using haemometer Sahli plano paralal method. On the other hand, the study by Valerian and colleagues [33] and Shrivastava et al [54] did not specify the cutoff and method of assessment used (just low hemoglobin). Demographic variables such as gender and age have been shown to be independently associated with hemoglobin deferral in a study that made use of 715,000 blood donors. The study showed female gender possessing 11-times greater chance of being deferred than their male counterpart whereas increasing age in males (effect not noted in females) was linked with 1.5-times greater chance of deferral per decade [60, 61]. Smoking status has also been documented to elevate Hb and consequently lower deferral due to Hb owing to the compensatory mechanism to the resulting chronic hypoxia created by carboxyhemoglobin [62]. Hemoglobin increment of 0.26 g/dL has been observed in donors who smoked 10 or less cigarettes per day, 0.59 g/dL increment in Hb has been observed in those who smoked more than 10 cigarettes per day, suggesting that Hb elevation associated with smoking may lead to low Hb deferrals [61, 63]. Despite the fact that a shortened inter-donation interval is associated with increased risk for deferral [64], increasing donation frequency is associated with decreased risk of deferral [65].

Comparatively, like most centers in Nigeria, the number of donations recorded in this study is high. Component separation is a scarce practice in the studied centre (mostly whole blood donation) as evident in previous study in the study center [1].

## Limitations

The results of this study need to be interpreted in view of a number of limitations. Though University of Calabar Teaching Hospital is the major health facility in the study area, extrapolation of the study result to the entire population need to be done with caution. More so, the study took a retrospective approach, missing data and selection bias common in retrospective studies may not be ruled out.

## Conclusion

Our study showed a deferral rate relatively low compared to most Nigerian studies. Hepatitis B and anemia constituted the major reasons for deferral in our study center. Stringent donor selection criteria have immense potential for protecting the donor and the recipient. However, there is need to balance between the urge to be strict in screening protocol to protect recipient and the need not to engage in unnecessary deferral as studies have shown that deferral has a negative impact on future donor return, particularly first-time donors and those deferred for more than a year [66, 67]. This study clearly pointed out HBV as the commonest cause of deferral, hence, intervention program should be undertaken to reduce the infection rate in the population. More so, voluntary blood donation should be encouraged over remunerated donations.

## Data Availability

Datasets generated and analyzed in this study are available from the corresponding author on request.

## Abbreviations

dL: Decilitre
FHI: Family health international
g: Gram
Hb: Hemoglobin
HBsAg: Hepatitis B surface antigen
HBV: Hepatitis B virus
HCV: Hepatitis C virus
HIV: Human immunodeficiency virus
L: Litre
NACA: National agency for control of AIDS
PCV: Packed cell volume
PEPFAR: President’s emergency plan for AIDS relieve
TTIs: Transfusion transmissible infections
UNAIDS: Joint united nations program on HIV/AIDS
VDRL: Venereal disease research laboratory
WHO: World health organization
